# *In vitro* shoot proliferation and *in vitro* and *ex vitro* root formation of *Pyrus elaeagrifolia* Pallas

**DOI:** 10.3389/fpls.2015.00225

**Published:** 2015-03-31

**Authors:** Ahmet Aygun, Hatice Dumanoglu

**Affiliations:** ^1^Department of Horticulture, Faculty of Agriculture, Ordu University, OrduTurkey; ^2^Department of Horticulture, Faculty of Agriculture, Ankara University, AnkaraTurkey

**Keywords:** micropropagation, *Pyrus elaeagrifolia*, tissue culture, wild pear

## Abstract

Shoot-tip cultures of *Pyrus elaeagrifolia* Pallas, an important gene source for drought and chlorosis resistance in pear rootstock breeding, were established from a wild mature tree originated from seed. Murashige and Skoog basal medium supplemented with different concentrations of benzyladenine (BA) singly or in combination with auxin was used in the study. In the initial culture, the highest percentages (>80%) of shoot proliferation were obtained in the mediums supplemented with 9.0 μM BA and 0.5 μM indole-3-acetic acid. In the subcultures, the highest shoot proliferation rates were obtained in the medium containing 4.5 and 9.0 μM BA. The shoot proliferation rates ranged from 91.1 ± 2.4 to 96.4 ± 2.0% in the second subculture and from 76.7 ± 7.8 to 89.4 ± 3.3% in the third subculture. In the second subculture, the shoots grown on 9.0 μM BA without auxin produced the best proliferation (10.6 ± 1.6). For the *in vitro* rooting experiments, the highest rooting rate (54.2 ± 10.4%), root length (10.5 ± 2.4 mm), and root number (2.5 ± 0.6) were obtained from 10 days dark treatment on the medium containing half strength of macronutrients supplemented with 5 μM indole-3-butyric acid (IBA). For the *ex vitro* rooting experiments, shoot rooting was significantly influenced by 10 mM IBA applied as quick-dip method. The percentage of rooting was 55 ± 9.6% and root number was 1.8 ± 0.3 at this concentration.

## Introduction

Iron-chlorosis in pears is a widespread problem, particularly in regions with calcareous soils (Dolcet-Sanjuan et al., 2004). *Pyrus elaeagrifolia* Pallas is one of the gene sources used to improve rootstock tolerance to drought and chlorosis (Lombard and Westwood, 1987). Thus, Claveria et al. (2010) used interspecific crosses including *Pyrus amygdaliformis, P. amygdaliformis* ssp. persica, *P. elaeagrifolia*, and *P. communis* ssp. cordata in developing resistant rootstocks to iron chlorosis. According to Lombard and Westwood (1987), drought tolerance and nitrogen, phosphorus, iron, boron, and zinc uptake from soil of pear scion cultivar by *P. elaeagrifolia* are at high levels. Moreover, this species is resistant to black end, pear decline, and wooly pear aphid. In Turkey, *P. elaeagrifolia* seedlings are used as rootstock for *P. communis* (Lombard and Westwood, 1987; Bell et al., 1996). In general, older wild trees of the species are grafted by pear scions (Koksal et al., 2002). Thus, healthy pear trees could be grown in arid soils with pH 7.5–8. However, seedlings of this species display high genetic variation. For these reasons, vegetative clonal propagation techniques could be used for selected *P. elaeagrifolia* genotypes as rootstocks.

*Pyrus elaeagrifolia* originated from Turkey, Russia, and southeast Europe (Lombard and Westwood, 1987; Bell et al., 1996). There are many types of this species in the Anatolian region of Turkey. The selection of clonal rootstocks from these dwarf or semi-dwarf genotypes, which are tolerant to chlorosis, and drought, resistant to diseases and pests, is very important in pear breeding. Similar to most fruit trees and nut species, reproduction of uniform copies of an original *P. elaeagrifolia* parent plant is possible by cuttings, layering, and micro-propagation. Among these techniques, only propagation by cutting has been studied in this species. Two types of *P. elaeagrifolia* have been propagated by softwood cuttings: the rooting percentages were determined as 11.4 and 43.8% (Dumanoglu et al., 1999). Micro-propagation allows fruit breeders to quickly multiply a new rootstock in a short time (Webster, 1995; Hartmann et al., 1997). Axillary bud multiplication is a widely used means of mass propagation of plants that are genotypically and phenotypically the same as the original plant from which they were produced (Evans, 1990). Results on micropropagation of *P. communis* (Cheng, 1979; Shen and Mullins, 1984; Rodriguez et al., 1991; Al-Maarri et al., 1994; Iglesias et al., 2004), *P. calleryana* (Berardi et al., 1993), *P. calleryana*, *P. betulaefolia* (Yeo and Reed, 1995), and *P. syriaca* (Shibli et al., 1997) have been reported by several researchers (Chevreau and Bell, 2005). However, micropropagation of *P. elaeagrifolia* has not been studied. In this study, we report *in vitro* shoot proliferation and *in vitro* and *ex vitro* root formation of *P. elaeagrifolia*.

## Materials and Methods

### Plant Material

In the present study, a wild mature tree originated from the seed of *P. elaeagrifolia* Pallas was used as plant material, and *in vitro* shoot-tip cultures were established from actively growing shoots.

### Explants Establishment and Shoot Proliferation Experiments

Murashige and Skoog (1962) basal medium containing 3% (w/v) sucrose and 0.7% (w/v) Difco Bacto agar was used in all experiments. The MS basal medium was supplemented with 0.3 μM gibberellic acid (GA_3_). For the initial culture and multiplication experiments, benzyladenine (BA; 0.0, 4.5, and 9.0 μM), in combination with indole-3-butyric acid (IBA; 0.0, 0.5, and 2.5 μM), and indole-3-acetic acid (IAA; 0.0, 0.5, and 2.5 μM) was used. All growth regulators were added to the media before autoclaving at 121°C for 20 min. The pH was adjusted to 5.7 before adding agar and autoclaving. All cultures were kept at 24 ± 1°C under a photoperiod of 16 h of cool white fluorescent light (35 μmol⋅m^-2^⋅s^-1^).

For explants establishment, shoot tips of 2 cm length were washed in running tap water for 5 min and surface sterilized in a solution of sodium hypochlorite (3% active chlorine) with 0.1% (v/v) Tween 20 for 15 min and rinsed three times with sterile distilled water. The explants of shoot tips (≈1 cm) were cultured in glass tubes (12 cm× 2.5 cm) containing 10 ml of MS medium supplemented with BA combinations of IBA and IAA. After 4 weeks in culture, the percentage of explants forming shoots and the number of shoots that were >1 cm long was recorded.

*In vitro* shoots to be used as explants for multiplication experiments should be in sufficient number. For this reason, the shoots (>1 cm) from the initial cultures were subcultured on MS medium supplemented with 9.0 μM BA for 4 weeks. Then, the shoots proliferated on this medium were subcultured in glass flasks (250 ml) containing 50 ml of MS medium supplemented with combinations of BA and IBA or IAA at 4 week intervals, three times. Every time, the shoots were subcultured on fresh medium of the same composition as the previous subculture. The percentage of shoot multiplication and the number of shoots (>1 cm) per proliferated shoot were only recorded at the end of the second and third subcultures on the MS medium supplemented with different BA × auxin combinations. The data of the first subculture was not evaluated since the explants used in this subculture were taken from the cultures on the MS medium supplemented with 9.0 μM BA.

The experiments with three factors (BA concentration, auxin concentration, and auxin type) were set up as a completely randomized design with factorial combinations of BA (0.0, 4.5, and 9.0 μM) and IBA (0.0, 0.5, and 2.5 μM) or IAA (0.0, 0.5, and 2.5 μM). Each treatment in the initial culture included 12 replications (tubes). The shoot multiplication experiment consisted of three replications (flasks) with five shoots in each treatment.

### Rooting Experiments

The rooting experiments were conducted *in vitro* and *ex vitro* conditions with micropropagated shoots approximately 1–2 cm long obtained from the fifth subcultures. The MS basal medium containing half strength of macronutrients supplemented with 3% (w/v) sucrose and 0.7% (w/v) Difco Bacto agar was used for *in vitro* rooting experiment. The effects of various auxin treatments, which were basal medium with no growth regulators (control), naphthaleneacetic acid (NAA; 5 and 10 μM), and IBA (5 and 10 μM) in basal medium, 10 – second dip in IBA (10 and 20 mM) and dissolved in 50% ethanol, were tested in light or darkness for 10 days followed by light. A two-factor (auxin × darkness) experiment was set up as a completely randomized design with factorial combinations of auxin and darkness treatments. Each treatment included 24 replications (tubes).

For *ex vitro* rooting experiments, microcuttings were quick-dipped in solutions of 0, 10, 20, 30, or 40 mM IBA in 50% ethanol. These cuttings were inserted into plastic boxes (10 cm × 8 cm × 5 cm) containing perlite. The box was sealed with plastic film to prevent desiccation. The humidity in the boxes was gradually reduced by cracking open the seals. The experiment was a one-factor (auxin treatments) completely randomized design, consisted of four replications (boxes) with five microcuttings in each treatment. The cultures were grown at 24 ± 1°C under 16-h photoperiod with 35 μmol⋅m^-2^⋅s^-1^ supplied by cool-white fluorescent bulbs for both *in vitro* and *ex vitro* treatments.

The data was collected after 45 days for *in vitro* and 75 days for *ex vitro* experiment. The percentage of rooted shoots, the number of roots, and average root length per rooted shoot were determined. The calluses were rated on a scale of 0–4 (0 = no callus, 1 = very small, 2 = small, 3 = medium, 4 = large) per shoot.

The plantlets were transplanted to the pots containing an autoclaved mixture of peat (70%), perlite (12%), sand (12%), and orchard soil (6%) and the pots were covered by plastic film. The plastic film was particularly opened after 3 weeks in the growth room.

### Statistical Analysis

Multifactorial variance analysis (ANOVA) was performed on the data by Minitab software (MINITAB Inc.). Means were compared by Duncan’s multiple range test (*P* < 0.05). Before the analysis, Arcsin transformations were used for the percentage data.

## Results

### Shoot Proliferation

In the initial culture, the interaction effects of BA × auxin concentration and auxin type × auxin concentration on shoot proliferation rate were significant (**Table 1**). Almost at all concentrations of auxin, 4.5, and 9.0 μM BA produced the highest shoot proliferation rates. On the other hand, shoot proliferation increased with 0.0 and 0.5 μM concentrations of IAA and 2.5 μM concentration of IBA. In the initial culture, the highest percentages (>80%) of shoot proliferation were obtained from the medium supplemented with 9.0 μM BA and 0.5 μM IAA (**Table 1**) In the second and the third subcultures, the shoot multiplication rate was significantly influenced by BA concentrations with the highest obtained from 4.5 to 9.0 μM BA concentrations. The shoot multiplication rates ranged from 91.1 ± 2.4 to 96.4 ± 2.0% and from 76.7 ± 7.8 to 89.4 ± 3.3% in the second and third subculture, respectively.

**Table 1:** Effect of benzyladenine (BA), indole-3-butyric acid (IBA), and indole-3-acetic acid (IAA) on the percentage of shoot proliferation in *Pyrus elaeagrifolia* Pallas.

Effects	Initial culture			Second subculture			Third subculture		
	Proliferation (%)		*P*	Proliferation (%)		*P*	Proliferation (%)		*P*
BA concentration (concn)						0.000			0.000
0.0 μM				1.8 ± 1.8	b		0.0 ± 0.0	b	
4.5 μM				91.1 ± 2.4	a		76.7 ± 7.8	a	
9.0 μM				96.4 ± 2.0	a		89.4 ± 3.3	a	
Auxin type									
IBA									
IAA									
Auxin concn									
0.0 μM									
0.5 μM									
2.5 μM									
Significant interaction effects									
BA concn × auxin concn			0.042						
0.0 μM × 0.0 μM	50.0 ± 10.4	bc							
0.0 μM × 0.5 μM	50.0 ± 10.4	bc							
0.0 μM × 2.5 μM	52.2 ± 10.6	bc							
4.5 μM × 0.0 μM	79.2 ± 8.5	ab							
4.5 μM × 0.5 μM	68.2 ± 10.2	ab							
4.5 μM × 2.5 μM	75.0 ± 9.0	ab							
9.0 μM × 0.0 μM	58.3 ± 10.3	abc							
9.0 μM × 0.5 μM	83.3 ± 7.8	a							
9.0 μM × 2.5 μM	37.5 ± 10.1	c							
Auxin type – auxin concn			0.001						
IBA – 0.0 μM	55.6 ± 8.4	bc							
IBA – 0.5 μM	51.4 ± 8.6	bc							
IBA – 2.5 μM	68.6 ± 8.0	ab							
IAA – 0.0 μM	69.4 ± 7.8	ab							
IAA – 0.5 μM	82.9 ± 6.5	a							
IAA – 2.5 μM	41.7 ± 8.3	c							

*Mean separation within columns followed by the same letter are not significantly different at *P* < 0.05 by Duncan’s multiple range test.*

**FIGURE 1:**
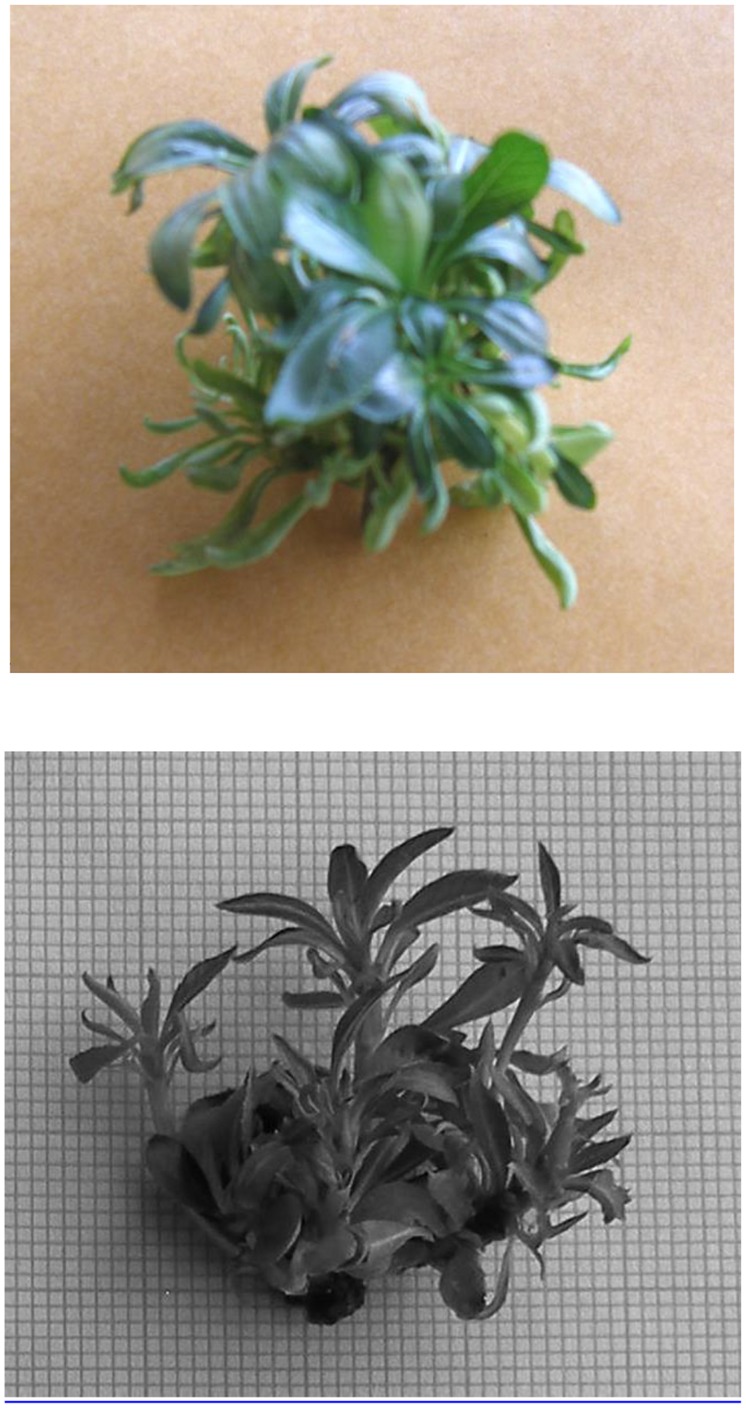
**Shoot proliferation in *Pyrus elaeagrifolia* on MS medium supplemented with 4.5 μM benzyladenine (BA) and 0.3 μM gibberellic acid (GA_3_)**.

Benzyladenine concentration × auxin type × auxin concentration interaction effect on the number of shoots per explant was significant in the initial culture (**Table 2**). The highest shoot proliferation (1.7 ± 0.2) was obtained from the medium containing 4.5 μM BA to 2.5 μM IBA. The mean shoot proliferation was not high for explants at this stage, whereas it highly increased in the second subculture (**Table 2**). The interaction of BA × auxin concentration was significant and the shoots grown on 9.0 μM BA without auxin produced the best shoot proliferation (10.6 ± 1.6). The highest number of shoots was recorded at 9.0 μM BA in the third subculture (**Figure 1**). Multiplication was significantly influenced by BA concentrations in this subculture.

**Table 2:** Effect of benzyladenine (BA), indole-3-butyric acid (IBA), and indole-3-acetic acid (IAA) on means of shoot proliferation in *Pyrus elaeagrifolia* Pallas.

Effects	Initial culture			Second subculture			Third subculture		
	Shoots per explant (no.)		*P*		Shoots per explant (no.)	*P*	Shoots per explant (no.)		*P*
BA concentration (concn)									0.001
0.0 μM							–		
4.5 μM							4.8 ± 0.3	b	
9.0 μM							6.7 ± 0.4	a	
Auxin type									
IBA									
IAA									
Auxin concn									
0.0 μM									
0.5 μM									
2.5 μM									
Significant interaction effects									
BA concn × auxin concn						0.039			
0.0 μM × 0.0 μM				–					
0.0 μM × 0.5 μM				–					
0.0 μM × 2.5 μM				–					
4.5 μM × 0.0 μM				6.0 ± 0.4	b				
4.5 μM × 0.5 μM				5.9 ± 0.4	b				
4.5 μM × 2.5 μM				5.6 ± 0.7	b				
9.0 μM × 0.0 μM				10.6 ± 1.6	a				
9.0 μM × 0.5 μM				5.1 ± 0.9	b				
9.0 μM × 2.5 μM				7.7 ± 1.3	b				
Auxin type – auxin concn									
IBA – 0.0 μM									
IBA – 0.5 μM									
IBA – 2.5 μM									
IAA – 0.0 μM									
IAA – 0.5 μM									
IAA – 2.5 μM									
BA concn × auxin type × auxin concn			0.007						
0.0 μM × IBA × 0.0 μM	1.2 ± 0.2	bc							
0.0 μM × IBA × 0.5 μM	1.0 ± 0.0	c							
0.0 μM × IBA × 2.5 μM	1.3 ± 0.2	b							
0.0 μM × IAA × 0.0 μM	1.0 ± 0.0	c							
0.0 μM × IAA × 0.5 μM	1.0 ± 0.0	c							
0.0 μM × IAA × 2.5 μM	1.0 ± 0.0	c							
4.5 μM × IBA × 0.0 μM	1.3 ± 0.2	b							
4.5 μM × IBA × 0.5 μM	1.7 ± 0.2	a							
4.5 μM × IBA × 2.5 μM	1.1 ± 0.1	c							
4.5 μM × IAA × 0.0 μM	1.0 ± 0.0	c							
4.5 μM × IAA × 0.5 μM	1.0 ± 0.0	c							
4.5 μM × IAA × 2.5 μM	1.0 ± 0.0	c							
9.0 μM × IBA × 0.0 μM	1.0 ± 0.0	c							
9.0 μM × IBA × 0.5 μM	1.0 ± 0.0	c							
9.0 μM × IBA × 2.5 μM	1.0 ± 0.0	c							
9.0 μM × IAA × 0.0 μM	1.0 ± 0.0	c							
9.0 μM × IAA × 0.5 μM	1.0 ± 0.0	c							
9.0 μM × IAA × 2.5 μM	1.0 ± 0.0	c							

*Mean separation within columns followed by the same letter are not significantly different at *P* < 0.05 by Duncan’s multiple range test.*

### Rooting

The highest rooting percentages were obtained as 54.2 ± 10.4 and 55 ± 9.6% from *in vitro* and *ex vitro* experiments, respectively. At both *in vitro* and *ex vitro* conditions, root formation was not observed in controls, the media without growth regulators. Auxin × darkness interaction was significant for *in vitro* rooting experiments (**Table 3**). The highest rooting rate (54.2 ± 10.4%) and root length (10.5 ± 2.4 mm) were obtained from 10 days dark treatment on the medium supplemented with 5 μM IBA. However, root numbers per rooted shoot on medium with 5 μM IBA were statistically similar at both light (2.4 ± 0.9) and dark (2.5 ± 0.6) treatments. NAA did not promote *in vitro* root formation. Generally, the shoots cultured on the medium supplemented with high levels of IBA (10 μM) or quick-dipped in 10 or 20 mM IBA solutions at both dark and light, produced larger callus than the other media. Callus degree (0–4) of *in vitro* micro-cuttings ranged from 0.0 to 3.8 ± 0.1 (**Table 3**).

**Table 3:** Effect of auxin and darkness on *in vitro* rooting of shoots in *Pyrus elaeagrifolia* Pallas.

Effects	Shoots rooting (%)		*P*	Roots per shoot (no.)		*P*	Root length (mm)		*P*	Callus (0–4)		*P*
Auxin												
None												
5 μM naphthaleneacetic acid (NAA)												
10 μM NAA												
5 μM IBA												
10 μM IBA												
10 mM IBA, dip												
20 mM IBA, dip												
Darkness												
Light												
Dark (10 days)												
Significant interaction effects												
Auxin × darkness			0.002			0.008			0.001			0.000
None × light	0.0 ± 0.0	d		–			–			0.0 ± 0.0	g	
5 μM NAA × light	0.0 ± 0.0	d		–			–			0.3 ± 0.1	fg	
10 μM NAA × light	0.0 ± 0.0	d		–			–			0.7 ± 0.1	ef	
5 μM IBA × light	29.2 ± 9.5	bc		1.5 ± 0.6	ab		5.1 ± 1.7	b		1.0 ± 0.0	de	
10 μM IBA × light	0.0 ± 0.0	d		–			–			3.8 ± 0.1	a	
10 mM IBA, dip × light	0.0 ± 0.0	d		–			–			2.3 ± 0.3	c	
20 mM IBA, dip × light	33.3 ± 9.8	b		2.4 ± 0.9	a		4.5 ± 1.7	b		1.5 ± 0.3	d	
None × dark	0.0 ± 0.0	d		–			–			0.0 ± 0.0	a	
5 μM NAA × dark	0.0 ± 0.0	d		–			–			0.6 ± 0.1	ef	
10 μM NAA × dark	0.0 ± 0.0	d		–			–			1.0 ± 0.1	de	
5 μM IBA × dark	54.2 ± 10.4	a		2.5 ± 0.6	a		10.5 ± 2.4	a		1.4 ± 0.2	d	
10 μM IBA × dark	0.0 ± 0.0	d		–			–			3.7 ± 0.1	a	
10 mM IBA, dip × dark	16.7 ± 7.8	cd		0.3 ± 0.1	c		4.2 ± 2.0	b		2.4 ± 0.3	c	
20 mM IBA, dip × dark	12.5 ± 6.9	d		0.6 ± 0.4	bc		1.2 ± 0.7	c		3.1 ± 0.2	b	

*Mean separation within columns followed by the same letter are not significantly different at *P* < 0.05 by Duncan’s multiple range test.*

In *ex vitro* rooting experiments, the shoot rooting was significantly influenced by 10 mM concentration of IBA in the quick-dip method (**Table 4**). The highest rooting percentage (55 ± 9.6%) and root numbers per rooted shoot (1.8 ± 0.3) were obtained from this concentration at the end of 75 days. The quick-dip in IBA treatments did not produce significant effect on root length and callus degree. The mean root length, measured between 17.6 ± 10.4 and 28.9 ± 7.0 mm, was higher than that obtained from *in vitro* experiments. One of the reasons for longer roots on micro-cuttings was the duration of the stay in perlite (75 days). Despite high concentration of IBA such as 40 mM, the callus degree of micro-cuttings ranged from 0.0 to 0.1 ± 0.03 (**Table 4**).

**Table 4:** Effect of quick-dip treatment in solutions of indole-3-butyric acid (IBA) on *ex vitro* rooting of shoots in *Pyrus elaeagrifolia* Pallas.

Effects	Shoots rooting (%)	*P*	Roots per shoot (no.)	*P*	Root length (mm)	*P*	Callus (0–4)	*P*
		0.005		0.004		0.054		0.382
None	0.0 ± 0.0	b	–		–		0.05 ± 0.03	
10 mM IBA quick – dip	55.0 ± 9.6	a	1.8 ± 0.3	a	28.9 ± 7.0		0.00 ± 0.00	
20 mM IBA quick – dip	25.0 ± 15.0	b	0.7 ± 0.5	b	17.6 ± 10.4		0.05 ± 0.05	
30 mM IBA quick – dip	20.0 ± 11.5	b	0.6 ± 0.4	b	17.9 ± 10.5		0.00 ± 0.00	
40 mM IBA quick – dip	0.0 ± 0.0	b	–		–		0.00 ± 0.00	

*Mean separation within columns followed by the same letter are not significantly different at *P* < 0.05 by Duncan’s multiple range test.*

## Discussion

In the present study, the shoot tip-culture from a mature tree of *P. elaeagrifolia* was successfully established on MS medium supplemented with some growth regulators. Tissue contamination and browning were not observed during the initial cultures and subcultures. *In vitro* shoot proliferation of *P. elaeagrifolia* was affected by BA concentrations (4.5 and 9.0 μM) in both initial cultures and subcultures. It was reported that BA concentrations of 3.3 to 20 μM produced successful multiplication of *P. communis* (Singha, 1982; Shen and Mullins, 1984; Baviera et al., 1989; Dolcet-Sanjuan et al., 1990; Moretti et al., 1991; Rodriguez et al., 1991; Yeo and Reed, 1995; Iglesias et al., 2004), *P. calleryana* (Dolcet-Sanjuan et al., 1990; Rossi et al., 1991; Berardi et al., 1993; Yeo and Reed, 1995), *P. amygdaliformis* (Dolcet-Sanjuan et al., 1990), *P. betulifolia* (Dolcet-Sanjuan et al., 1990; Yeo and Reed, 1995), and *P. syrica* (Shibli et al., 1997). Generally, researchers used BA alone in micro-propagation of *Pyrus* species and very little information is available about the effect of auxins on multiplication in this genus. Yeo and Reed (1995) examined the combinations of BA with IBA and NAA in multiplication of three rootstock selections of *P. calleryana*, *P. betulifolia*, and *P. communis*. The results revealed that IBA and NAA concentrations of >0.5 or 1 μM inhibited shoot multiplication in all three genotypes. Bhojwani et al. (1984) reported that shoots of *P. pyrifolia* were multiplied on MS medium supplemented with 6.7 μM BA and 0.1 μM NAA. Our results on IBA are in agreement with those of Yeo and Reed (1995). In the present study, a combination of high concentrations of BA (9.0 μM) and auxin (2.5 μM) inhibited shoot proliferation of *P. elaeagrifolia* in the initial culture. In addition, heavy callusing of the basal end and hyperhydricity of the *in vitro* shoots occurred at >9.0 μM BA and >2.5 μM IAA or IBA (data not shown). There is no information on the effects of IAA in *Pyrus* species. Our study indicated that IAA at 0.5 μM produced the highest percentage of shoot proliferation in the initial culture. According to this result, IAA is important for shoot proliferation in *P. elaeagrifolia*.

Both *in vitro* and *ex vitro* rooting of micro-propagated shoots of *P. elaeagrifolia* were only accomplished by auxin treatments. Such results were found in some other *Pyrus* species and woody plants (Bhojwani et al., 1984; Shen and Mullins, 1984; Baviera et al., 1989; McClelland et al., 1990; Berardi et al., 1991, 1993; Dolcet-Sanjuan et al., 1991; Moretti et al., 1991; Rodriguez et al., 1991; Rossi et al., 1991; Al-Maarri et al., 1994; Reed, 1995; Yeo and Reed, 1995; Shibli et al., 1997; Iglesias et al., 2004; Barros et al., 2005). In the current study, IBA at low concentration (5 μM) with dark treatment (10 days) stimulated *in vitro* root formation in micro-cuttings of *P. elaeagrifolia*. This result agrees with the fact that a dilute mineral medium with 0.1–10 μM IBA and NAA and an initial period of 7–10 days of dark incubation, improves rooting of pear *in vitro* shoots (Chevreau and Bell, 2005). We also used MS basal medium with half strength of macronutrients in the rooting experiment, but the root formation was not obtained on medium supplemented with NAA. However, Al-Maarri et al. (1994) reported that the best exogenous auxin for *in vitro* rooting of cultivars ‘Passe Crassane’ and ‘Williams’ pear seedlings was NAA at 1 μM. It was reported that root formation of the *Pyrus* species (*P. betulaefolia, P. communis, P. calleryana*, and *P. amygdaliformis*) was stimulated by exposure of shoots to high levels (10 or 32 μM) of IBA for 7 days followed by a passage on auxin-free medium (Dolcet-Sanjuan et al., 1990). Our result showed that IBA is effective auxin type for rooting of micro-cuttings in *P. elaeagrifolia*. Similarly, especially for recalcitrant *Pyrus* genotypes, treatment with 10 μM IBA during 1 or 3 weeks in darkness was suggested (Reed, 1995; Yeo and Reed, 1995), but IBA concentrations lower than 10 μM were not tested in the studies. Berardi et al. (1993) found that *in vitro* rooting in *P. calleryana* was promoted by auxins, mainly by NAA (2.7 μM). Shibli et al. (1997) reported that IBA, IAA, and NAA induced *in vitro* rooting of *P. syrica* and a maximum of 72% rooting was achieved at 17 μM IAA. For *in vitro* rooting of Conference pear cultivar, IAA at 2.7 μM was found to be most appropriate (Baviera et al., 1989). But, Al-Maarri et al. (1994) reported that IAA was unfavorable for rooting in pear shoots.

As another treatment, Reed (1995) recommended that IBA or NAA dip treatment can be used for rooting pear genotypes of unknown rooting potential. But, the effect of 10 and 20 mM IBA dip procedures on *in vitro* rooting in *P. elaeagrifolia* was not as high as that of medium supplemented with 5 μM IBA in dark. However, Rodriguez et al. (1991) reported that *in vitro* rooting without callus formation in *P. communis* was achieved by immersing the basal end in 5 μM IBA solution for 1 min.

Successful *ex vitro* rooting can save time and reduce costs (Yeo and Reed, 1995). In our study, 10 mM IBA quick-dip treatment in *ex vitro* conditions stimulated rooting, but higher concentrations of IBA prevented root formation from micro-cuttings. However, Yeo and Reed (1995) reported that neither rooting nor survival occurred from *ex vitro* rooting treatment in any *Pyrus* rootstocks. In addition, *ex vitro* rooting was found to be unsuccessful for *P. syrica* when treating micro-cuttings with 0–100 μM IBA, IAA, or NAA for 1 h (Shibli et al., 1997). Al-Maarri et al. (1994) reported that *ex vitro* rooting was induced after cold storage pretreatment for 1 month followed by immersion for 1 h in NAA solutions (0.05–0.5 mM).

## Conclusion

We reported here an effective clonal propagation protocol by shoot-tip culture from a wild mature tree of *P. elaeagrifolia*. The combination of 9.0 μM BA and 0.5 μM IAA and 4.5, or 9.0 μM BA without auxin is recommended for the initial cultures and subcultures, respectively. Rooting of micro-cuttings was good on MS medium modified with half-strength of macronutrients with 5 μM IBA and 10 days dark treatment for *in vitro* or 10 mM IBA quick – dip treatment for *ex vitro*. Acclimatization of plantlets was achieved in the growth room for 3 weeks. Currently, we are using this protocol successfully for other *in vitro* studies on *P. elaeagrifolia*.

## Conflict of Interest Statement

The authors declare that the research was conducted in the absence of any commercial or financial relationships that could be construed as a potential conflict of interest.
